# Perioperative FLOT chemotherapy plus surgery for oligometastatic esophagogastric adenocarcinoma: surgical outcome and overall survival

**DOI:** 10.1186/s12893-020-01035-9

**Published:** 2021-01-13

**Authors:** Mira Runkel, Rasmus Verst, Julia Spiegelberg, Stefan Fichtner-Feigl, Jens Hoeppner, Torben Glatz

**Affiliations:** 1 Department of General - and Visceral Surgery, Medical Center, University of Freiburg, Hugstetterstrasse 55, 79106 Freiburg, Germany; 2 Present Address: Department of Surgery, University Medical Center Schleswig-Holstein, Campus Luebeck, Luebeck, Germany; 3 Present Address: Department of Surgery, Marien Hospital Herne, Ruhr-University Bochum, 44625 Herne, Germany

**Keywords:** Esophageal cancer, Gastric cancer, Adenocarcinoma, Perioperative chemotherapy, Oligometastases

## Abstract

**Background:**

Guidelines do not recommend surgery for patients with oligometastatic disease from esophagogastric adenocarcinoma (EGAC), although some studies suggest a more favorable survival. We analyzed the outcome of oligometastatic EGAC receiving FLOT chemotherapy followed by surgery.

**Methods:**

The data of patients with either pre-therapeutic, post-neoadjuvant or intraoperative clinical diagnosis of oligometastatic EGAC were extracted from a prospective database of the 2009–2018 treatment period. 48 consecutive patients were identified with oligometastatic disease, who underwent perioperative chemotherapy plus surgery. We retrospectively analyzed surgical outcome and overall survival.

**Results:**

The overall 5-year survival was 18%. 12 patients (25%) with pre-therapeutic oligometastatic EGAC, who had no histologic vital tumor evidence of metastases after surgery had a survival rate of 48% compared to an 11% 5-year survival rate of 36 patients (75%), who had histologic vital tumor metastatic evidence after FLOT chemotherapy and surgical resection (p = 0.012). The survival rates after R0, R1 and R2 (non-resected metastases) resection were 21% (n = 33), 0% (n = 4) and 17% (n = 11), respectively (p = 0.273).

**Conclusion:**

Oligometastatic EGAC is associated with poor overall survival even after complete resection of all tumor manifestations. The subgroup of patients with a complete histologic response of metastatic lesions to neoadjuvant FLOT shows 5-year survival rates similar to non-metastatic EGAC.

*Trial registration* Not applicable.

## Background

Different randomized controlled trials (RCT) have demonstrated superior results of perioperative chemotherapy plus surgery for non-metastatic esophagogastric cancers (EGAC) compared to surgery alone [1–4]. The chemotherapy of choice in Europe is the FLOT regime, consisting of Docetaxel 50 mg/m^2^, Oxaliplatin 85 mg/m^2^, Leucovorin 200 mg/m^2^ and 5FU 2600 mg/m^2^. FLOT is associated with overall better survival compared to ECF/ECX regimes [5]. One RCT showed survival rates of 50 months for perioperative FLOT therapy vs 35 months for patients receiving ECF/ECX regimes [5]. Perioperative chemotherapy has been adopted by national and international guidelines for locally advanced but not for metastatic EGAC [3, 6] for which palliative chemotherapy is recommended. There is, however, great uncertainty about the best management for patients with oligometastatic EGAC. Oligometastastic disease is defined as less than five distant, potentially resectable metastases, eg liver metastases and limited peritoneal carcinomatosis. It is used to described a state between limited and disseminated metastatic disease, with the potential intention of curative treatment [7]. The evidence is low with some retrospective studies suggesting prolonged survival after surgical resection of metastases from EGAC tumors [8–10]. The ongoing RENAISSANCE trial, a multicenter RCT, comparing the effect of chemotherapy alone vs chemotherapy followed by surgery for patients with oligometastatic EGAC is anticipated to shed light on the best treatment modality [10]. As we await the results, we conducted an analysis of our prospective database, evaluating the outcome and 5-year survival of patients with oligometastatic EGAC, who received perioperative FLOT chemotherapy plus surgery of the primary tumor and its metastases.

## Methods

Between June 2009 and April 2018 277 patients had locally advanced EGAC, who were treated with FLOT chemotherapy and subsequent surgery at the Medical Center of the University of Freiburg. Out of these 277 patients, 48 patients with oligometastatic disease, including potentially resectable peritoneal carcinomatosis, underwent perioperative FLOT chemotherapy followed by surgery. The majority of patients with metastatic disease received palliative chemotherapy alone, and were not treated by our department. These patients were not included in our analysis. The metastases were found either at the time of initial diagnosis (cM1), during post-neoadjuvant staging investigations (ycM1), or found intraoperatively. As 9 patients were lost to follow up, a total of 220 patients showed no metastases at time of diagnosis (cM0). Data had been prospectively collected and retrospectively analyzed in this study. Patient demographics, pre- and postoperative tumor stages, histopathological regression (HPR), perioperative complications and administration of perioperative chemotherapy were correlated with overall survival of the patients.

Complications were classified according to Clavien Dindo [11] and tumor regression according to Becker et al. [12]. Survival data was obtained from the cancer registry of the Cancer Centre of our Medical Center. Inclusion in the cancer registry required informed consent, which was obtained from all patients. The study was approved by the Medical Ethics Committee of the University of Freiburg (File number 253/19).

### Diagnostic work up and Staging

All patients with symptoms suggesting the presence of esophageal or gastric cancers were taken through diagnostic and staging work up according to German S3 guidelines [6]. This includes a thorough medical history and physical exam, as well as upper GI endoscopy with several biopsies, endoscopic ultrasound if technically possible and a CT Thorax/Abdomen to exclude distant metastases. Diagnostic laparoscopy with peritoneal biopsies and PET-CT scans were added in selected cases of suspected peritoneal carcinomatosis or distant metastases, otherwise distant metastases were diagnosed by staging CT. The management pathway was chosen according to TNM staging. For patients with locally advanced EGAC (pT3 or pT4), re-staging was carried out after the completion of neoadjuvant chemotherapy, in order to plan surgical management.

### Chemotherapy and surgical resection

Perioperative chemotherapy consists of four cycles prior to surgery (over 8 weeks) and further four cycles post-surgery, with each cycle lasting 2 weeks. The FLOT regime consists of infusions of 5-FU 2600 mg/m^2^ (24 h), leucovorin 200 mg/m^2^ (2 h), oxaliplatin 85 mg/m^2^ (2 h) and docetaxel 50 mg/m2 (1 h) every 2 weeks [5].

Surgery was usually carried out between 4 and 6 weeks after the completion of the neoadjuvant cycles of chemotherapy, with few selected patients undergoing surgery at a later point in time. Surgery was chosen according to tumor location and size. Routinely, esophagectomy plus proximal gastrectomy with two-field lymphadenectomy was performed for esophageal or junctional adenocarcinoma (AEG I + II), whilst patients with AEG III tumors (in some selected cases also AEG II underwent transhiatal extended gastrectomy with lower mediastinal and modified DII-lymphadenectomy. Total or subtotal gastrectomy plus modified DII-lymphadenectomy was performed for patients with gastric cancer. In extended tumors, the surgical approach was adapted as necessary. Resectability of the primary and metastases depended on the location and was determined by an interdisciplinary team. It was then carried out accordingly e.g. as liver resection, adrenalectomy or peritonectomy for peritoneal carcinomatosis. Hyperthermic intraperitoneal chemotherapy (HIPEC) was additionally performed for patients with limited peritoneal metastases using Cisplatin (75 mg/m^2^) and Doxorubicin (15 mg/m^2^). No peritoneal lavage cytology was performed for patients in this cohort. Routine postoperative standard histopathological workup and staging was performed.

### Statistical analysis

Statistical analysis was performed using IBM SPSS statistics, Version 23. Categorical variables were put in absolute and relative frequencies; differences were evaluated by Chi-Square or Fisher’s exact test as appropriate. Quantitative values were expressed as medians with range and differences were measured using the Mann–Whitney-U test. Multivariate analysis was performed through forward logistic regression model, with relative risk and a 95% confidence interval. The Kaplan–Meier method was used to evaluate survival, with a long-rank test for the comparison of subgroups. A p-value < 0.05 was considered statistically significant.

## Results

### Early results

**Fig. 1:**
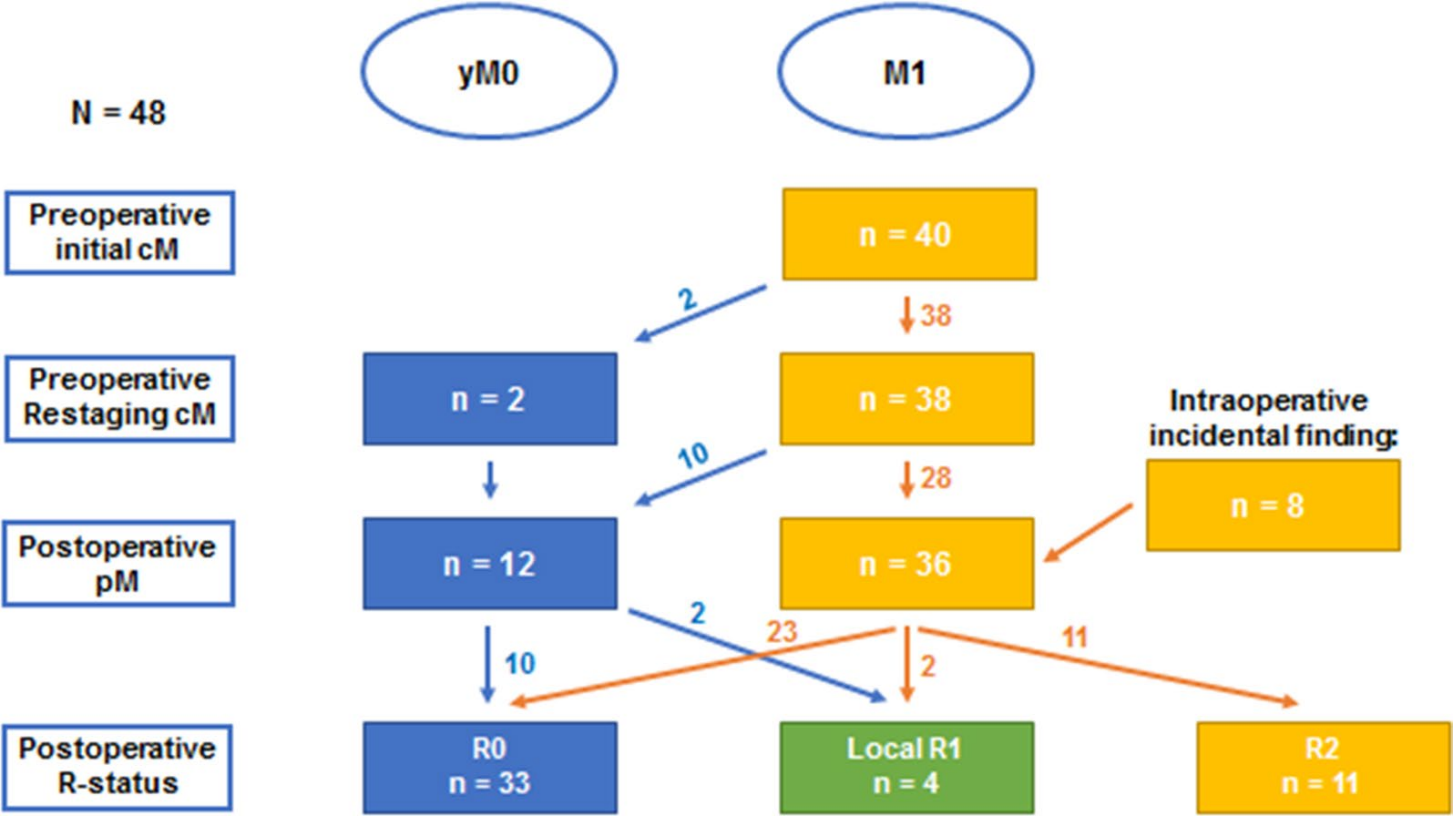
Flowchart of diagnosis and staging for patients with primary diagnosis of (oligometastatic) EGAC

Out of 48 patients, 31 patients (65%) were diagnosed with gastric cancer and 17 patients (35%) with esophageal adenocarcinoma. Median follow up was 13 months (11 months for deceased patients and 17 months for all others). All patients, except for one (pT2), had pT3 or pT4 tumors at initial staging. Comorbidities, including cardiac, pulmonary, renal and hepatic disease were present in 64% of patients (n = 31). Other patients’ characteristics are summarized in Table 1. 83% of patients completed four cycles of neoadjuvant FLOT chemotherapy (n = 40), whereas the adherence to postoperative chemotherapy was less, with only 31 patients (65%) completing their adjuvant treatment. Potentially resectable metastases were present in all patients either at the time of initial diagnosis or during preoperative staging. Figure 1 shows patient selection through a flowchart of diagnosis and staging (Fig. 1).

**Table 1:** Distribution of demographics for patients with oligometastatic EGAC

	Gastric carcinoma (n = 31)	Esophageal carcinoma (n = 17)	Total (n = 48)
Sex			
Female	11 (35%)	4 (23%)	15 (31%)
Male	20 (64%)	13 (76%)	33 (69%)
Age in years*	55.1 (33.6–81.1)	58.3 (30.2–80)	56.8 (30.2–81.2)
ASA classification			
ASA 1–2	17 (55%)	12 (70%)	29 (60%)
ASA 3–4	14 (45%)	5 (29%)	19 (39%)
BMI in kg/m^2^*	26.3 (18.0–41.7)	24.5 (20.5–38.1)	25.3 (18.0–41.7)

62% of patients (n = 30) had peritoneal carcinomatosis, from which 84% arose from gastric cancer (n = 26). Distant lymph node metastases (lymph nodes outside of DI-II resection area) were found in 7 patients, other distant metastases sites were hepatic (n = 7), adrenal (n = 3) and pulmonary (n = 1). Complete remission of metastases after preoperative chemotherapy was achieved in 12 patients (Fig. 1). Out of these 12 patients (ypM0), 8 did not show evidence of metastatic lesions, both during the preoperative staging and intraoperatively, and 4 patients with macroscopically suspected peritoneal carcinomatosis showed no histologic evidence of tumor cells after peritonectomy. Resection of metastases was performed if distant metastases could be detected intraoperatively and surgical resection seemed feasible. Thus, in 68% of patients (n = 33) simultaneous resection of metastases was performed. For 11 patients (23%) metastases were not feasible to resect, defined here as R2. HIPEC was additionally carried out in 15 patients with peritoneal metastases after complete cytoreductive surgery.

### Complications and length of stay

Postoperative complications occurred in 48% of patients (n = 23) [42% after gastrectomy (n = 13) and 59% after esophagectomy (n = 10)]. Surgical complications include anastomotic leaks (n = 2) wound infection (n = 5), chylothorax (n = 3) and haemorrhage (n = 2), whilst medical complications were mainly pulmonary, such as pleural effusions (n = 4), pneumonia (n = 8) and the need for reintubation (n = 2). 16 patients (33%) experienced complications of grade I-II and seven patients had major complications of grade III-V (15%). Two patients died after esophagectomy: one patient due to postoperative arrosive bleeding from the splenic artery and one due to rapid progressive pleural carcinomatosis, and one patient after gastrectomy, due to an anastomotic leak followed by septic shock. Average length of hospital stay was 12 days (range 7–94), with an average length of stay on ICU/IMC of 4 days (range 2–22 days). Treatment data is summarized in Table 2.

**Table 2:** Details of surgical intervention and postoperative treatment data including complications and recurrence

	Gastric carcinoma (n = 31)	Esophageal carcinoma (n = 17)	Total (n = 48)
Type of surgery			
Esophagectomy	1 (3%)	15 (88%)	16 (33%)
Gastrectomy	30 (97%)	2 (12%)	32 (66%)
HIPEC	14 (45%)	1 (6%)	15 (31%)
Additional resection			
None	10 (32%)	5 (29%)	15 (31%)
Peritoneum	16 (51%)	2 (12%)	18 (37%)
Distant lymph nodes	1 (3%)	3 (18%)	4 (8%)
Liver	1 (3%)	3 (18%)	4 (8%)
Adrenal	3 (10%)	0	3 (6%)
Multivisceral resection	0	4 (24%)	4 (8%)
Perioperative in-patient stay in days	11 (8–23)	14 (7–94)	12 (7–94)
Perioperative in intensive care in days	4 (2–11)	5 (3–22)	4 (2–22)
Perioperative complications			
Clavien Dindo	13 (42%)	10 (59%)	23 (48%)
I/II	9 (29%)	7 (41%)	16 (33%)
III/IV	3 (10%)	1 (6%)	4 (8%)
V	1 (3%)	2 (12%)	3 (6%)
Resection margin			
R0	21 (68%)	12 (70%)	33 (69%)
R1 (primary tumor)	3 (9%)	1 (6%)	4 (8%)
R2 (non-resected metastases)	7 (22%)	4 (23%)	11 (23%)
Postoperative residual tumor			
Local	3 (10%)	1 (6%)	4 (8%)
Peritoneal carcinomatosis	3 (10%)	2 (12%)	5 (10%)
Lymph nodes	1 (3%)	2 (12%)	3 (6%)
Distant metastasis	3 (10%)	0	3 (6%)
Recurrence	12 (39%)	7 (41%)	19 (40%)
Time of recurrence after surgery in months*	5,5 (1–15)	6,5 (1–10)	6 (1–15)
Type of recurrence			
Local	1 (8%)	0	1 (5%)
Local and distant	2 (17%)	2 (29%)	4 (21%)
Peritoneal carcinomatosis	4 (33%)	1 (14%)	5 (26%)
Hepatic metastasis	3 (25%)	3 (43%)	6 (32%)
Other type of metastasis	1 (8%)	1 (14%)	2 (11%)

### Overall survival

**Fig. 2:**
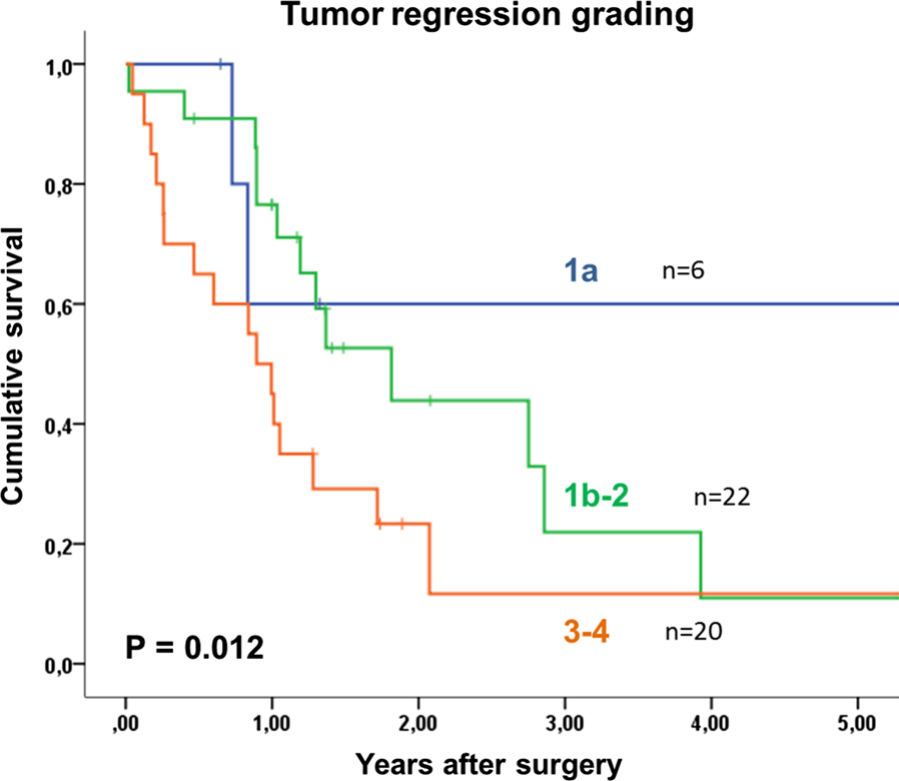
Kaplan Meier 5- year survival for patients with oligometastatic EGAC depending on postoperative tumor regression grading

**Fig. 3:**
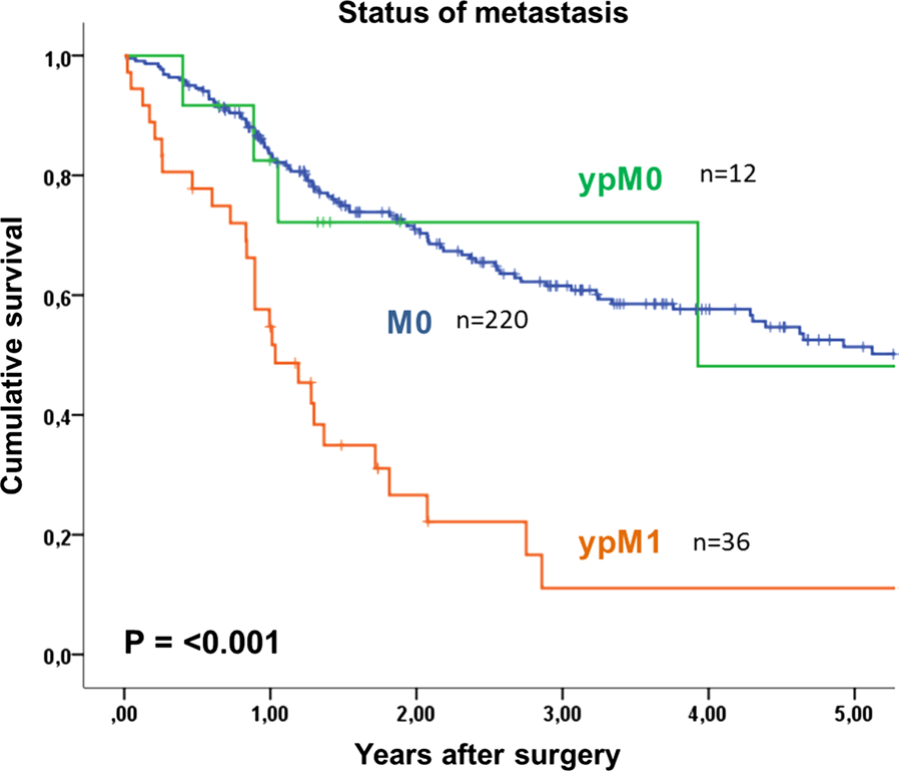
Kaplan Meier 5- year survival for patients with oligometastatic EGAC depending on status of metastases

**Fig. 4:**
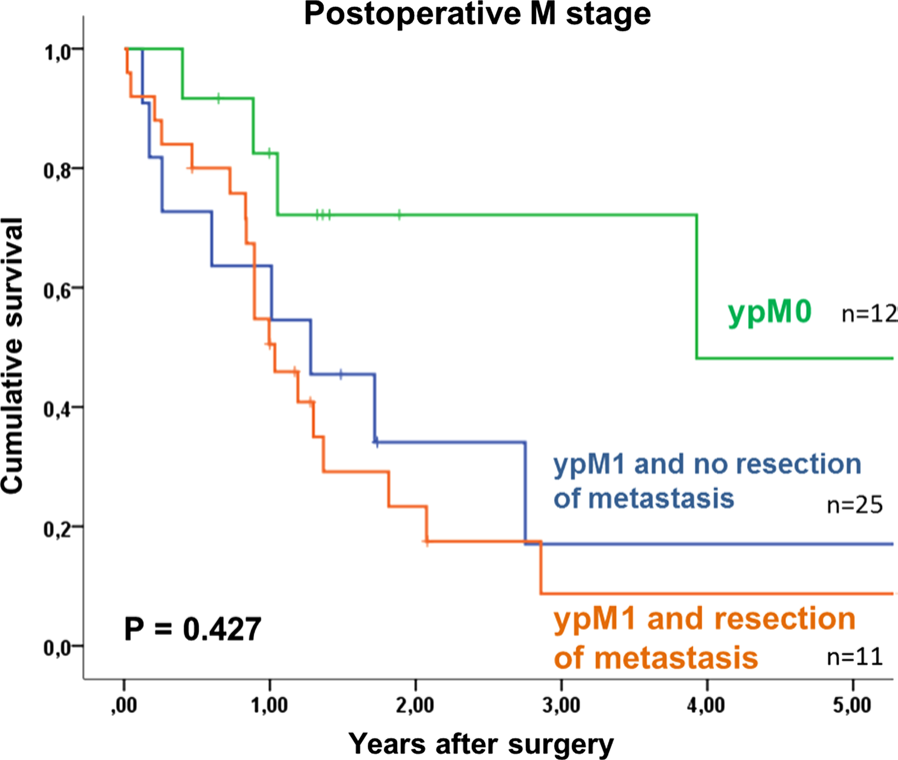
Kaplan Meier 5-year survival for patients with oligometastatic EGAC depending on postoperative M-stage

**Fig. 5:**
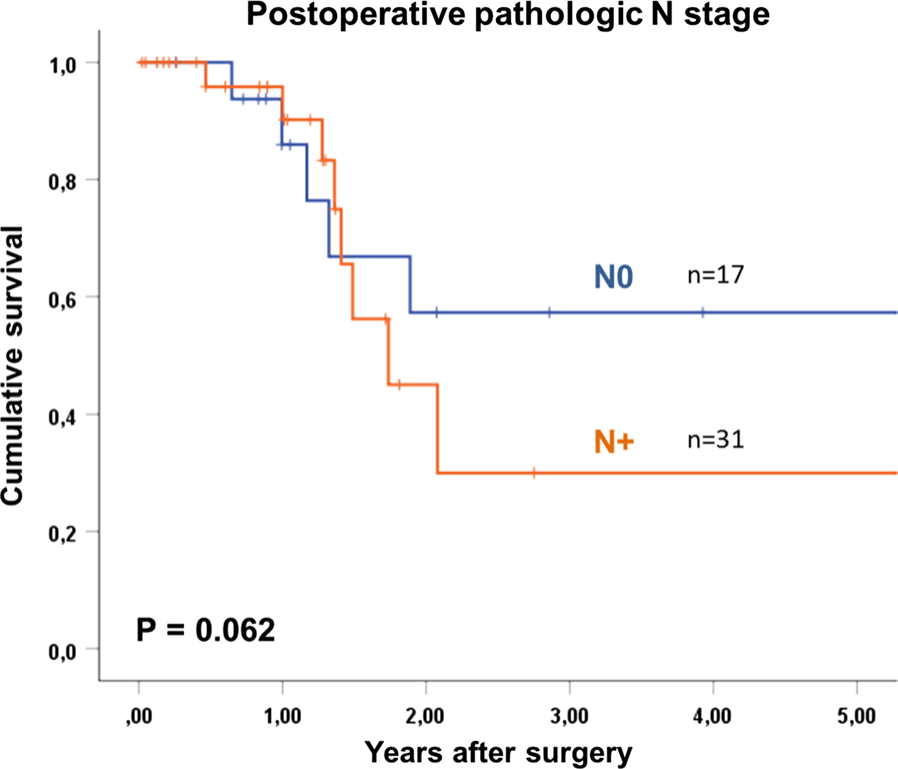
Kaplan Meier 5-year survival for patients with oligometastatic EGAC depending on postoperative pathologic N-staging

Overall 5- year survival of patients with oligometastatic EGAC was 18%, with a median survival of 15 months after surgery. Tumor recurrence occurred in 51% (19 of 37 patients) without residual macroscopic tumor after surgery, in a median time interval of 6 months (1–15). Most recurrences were distant metastases (peritoneal carcinomatosis n = 5, hepatic n = 6, multiple distant n = 1). Post-recurrence treatment was individualized to the patient and included surgery, radiotherapy, palliative chemotherapy or best supportive care. According to the treatment used, the rate of overall survival will differ. Patients with gastric cancer and esophageal adenocarcinoma had 5-year survival rates of 25% and 10% respectively (p = 0.213). Tumor regression grading according to Becker et al. [12], showed that with 1a regression (no residual tumor, n = 6), patients had a 60% survival at 5 years. Patients with regression grades of 1b and 2 only had 11% and grade 3 and above only 12% 5- year survival (p = 0.012, Table 3, Fig. 2). Median survival was 21 months and 9 months, respectively. The survival rate was shown to be 60% for patients with T0 staging, compared to 27% for T1/T2 and 0% for T3/T4 tumors (n = 6, n = 15, n = 27, respectively, p = 0.047). The most significant finding was demonstrated by the difference in 5-year survival rate between patients with non-detectable tumor postoperatively (ypM0) and patients with detectable oligometastases. Here, patients with postoperative ypM0 (n = 12) had a 48% 5-year survival rate, with a median survival of 47 months, in contrast to only 11% at 5 years for patients with detectable tumor cells (ypM1, n = 36), with a median of 12 months (p = 0.012). Furthermore, the overall survival of patients with ypM0 is comparable to patients without metastatic disease at primary diagnosis (cM0), with 48% and 51% respectively (p < 0.001; Fig. 3). There is no significant difference in overall survival between patients with resected metastases, and those without resection (9% vs 17% p = 0.427; Fig. 4). The location of metastases, resection margin or nodal status (Fig. 5) had no significant correlation for 5-year survival (p = 0.945, p = 0.273, p = 0.062). Postoperative T-stage showed an influence on 5-year survival (p = 0.047). Multivariate analysis regarding T-, N- and M-staging, R-status or regression grade showed that the absence of metastases (ypM0) is an independent predictor of overall survival (p = 0.018; RR 3.612, KI 12.46–10.470). Results are summarized in Table 3.

**Table 3:** Univariate analysis of factors influencing 5-year survival after resection of oligometastatic EGAC

	n	5-year-survival (%)	Median survival in years	p =
Total	48	18	1.3	
Sex				
Female	15	26	2.8	0.195
Male	33	16	1	
Age				
< 65	35	10	1.2	0.596
≥ 65	13	26	1.4	
ASA classification				
ASA 1–2	29	20	1.3	0.405
ASA 3–4	19	16	1	
Type of carcinoma				
Esophageal carcinoma	17	10%	1	0.213
Gastric carcinoma	31	24%	1.3	
Preoperative T stage				
T2	1	0	0.5	0.338
T3	29	14%	1.2	
T4	5	0	3.9	
Type of surgery				
Esophagectomy	16	0	1	0.099
Gastrectomy	32	30	1.3	
Resection margin				
R0	33	21	1.4	0.273
R1 (primary tumor)	4	0	0.4	
R2 (non-resected metastases)	11	17	1.3	
Tumor regression grading				
1a	6	60	Not reached	0.012
1b-2	22	11	1.8	
03-Apr	20	12	0.9	
Postop. pathologic T stage				
T0	6	60	Not reached	0.047
T1–T2	15	27	1.3	
T3–T4	27	0	1	
Postop. pathologic N stage				
N0	17	31	2.9	0.062
N+	31	11	1.2	
Type of metastasis				
Peritoneal carcinomatosis	30	11	1	0.945
Lymph nodes	7	43	1.3	
Hepatic	7	40	2.9	
Adrenal	3	0	0.9	
Pulmonary	1	0	1.7	
ypM0	12	48	3.9	0.427
ypM1 and complete resection of metastasis	25	9	1	
ypM1 and no complete resection of metastasis	11	17	1.3	
Status of metastasis				
No	12	48	3.9	0.012
Yes	36	11	1	
Adjuvant chemotherapy				
Yes	31	21	1.7	0.182
No	9	30	0.5

## Discussion

Multimodal treatment strategies have significantly improved the long-term results in the treatment of non-metastatic EGAC. Up to date, there is an ongoing debate about the best treatment option for EGAC with oligometastatic disease. Synchronous metastatic disease is seen in up to 14% of cases [13, 14]. Up to date, guidelines across Europe do not recommend multimodal treatment including surgery for patients with distant metastases, but rather recommend palliative chemotherapy [6, 15, 16]. The value of surgery for metastases, especially for liver metastases originating from colorectal cancer, has evolved over the last few years as it has been shown to improve long-term and disease-free survival, with 5 year survival rates of 25–40% [17, 18]. Thus, the option of surgery as a potential curative treatment is standardly offered to patients with hepatic metastatic colorectal disease. Furthermore, even though the addition of treatment modalities like cytoreductive surgery and HIPEC have been shown to prolong survival in selected patients with gastrointestinal and gynecological tumors there is in an ongoing debate about the best management of patients with metastatic disease [19–21]. Although some retrospective studies suggest an improved survival of patients with surgical resection of metastases for EGAC [22, 23], there is a lack of prospective, randomized evidence. There is currently an ongoing RCT comparing surgical intervention for oligometastatic disease to the effectiveness of palliative chemotherapy [10].

Comparing our survival data with the existing literature, certain consistencies can be identified. The median survival of all patients included in this study was 15 months, the data in the literature ranges from 13 to 31 months [9, 10, 13]. Most definitions of oligometastatic disease, however, do not include peritoneal carcinomatosis. A difference in 5-year survival is shown between lymphatic and hepatic metastases (43% and 40%) compared to peritoneal carcinomatosis of 11%, suggesting that results might differ according to which definition of oligometastases was used. Patients with peritoneal carcinomatosis are often only treated with best supportive care or palliative chemotherapy, with a median survival of 4 and 7 months, respectively [24]. Our data suggests median survival rates of 13 months for patients with peritoneal carcinomatosis and perioperative chemotherapy followed by surgery, with 15 patients receiving additional HIPEC. The median survival correlates with data found in the literature regarding HIPEC and gastric cancers quoting median survival of between 10 and 21 months [25–27]. Although some authors suggest an improved overall survival for patients with limited peritoneal metastatic disease and HIPEC, it is not introduced in national and international treatment guidelines for patients with EGAC [25, 26, 28–30].

Different studies suggest a significantly improved overall survival of patients with surgical resection of the primary EGAC and metastases [8–10, 13, 14, 31], although results from RCTs are still anticipated. Patients without any detectable metastases after perioperative chemotherapy and surgical resection (ypM0) had a similar overall survival to patients without any metastatic disease at primary diagnosis (48% and 52% at 5 years, respectively), demonstrating the effectiveness of good response to neoadjuvant chemotherapy. Median survival for patients with ypM0 of 47 months compares to median survival quoted in the literature for patients after FLOT therapy for locally advanced tumors of 50 months [5]. The phase 2 AIO-FLOT3 trial suggests better overall survival after resection compared to chemotherapy alone, quoting almost double the median survival (31.3 months vs 15.9 months) [31]. Patients selected for surgery of metastases had to show a chance of R0 resection of the primary and metastatic lesion at restaging, assuming a good response to preoperative chemotherapy. Metastases found intraoperatively suggest either progress of disease or lack of sensitivity of staging diagnostics. To the contrary an Asian RCT compared chemotherapy alone to a multimodal therapeutic approach and randomly assigned 175 patients with advanced gastric cancer and oligometastasis to either chemotherapy or gastrectomy followed by chemotherapy [32]. The multimodal treatment option of surgery followed by chemotherapy failed to show survival benefit over chemotherapy alone. Furthermore, complications associated with chemotherapy were higher in those who underwent gastrectomy beforehand, suggesting that additional surgery leads to decreased survival rates and higher rates of chemotherapy-associated complications [32]. Although our results suggest no additional benefit from surgical resection of the metastases, the comparison to the Asian trial is difficult. All our patients received neoadjuvant FLOT chemotherapy followed by resection; a comparison to chemotherapy alone was not made.

Similar to our results, a large retrospective analysis of 5185 patients did not show a survival benefit of simultaneous resection of metastases compared to resection of the primary alone [33]. Prognostic factors were pT- staging, regression grading and type of recurrence. Although some studies suggest significant influence of age, gender, sex, tumor location and nodal stage [2, 8, 13, 34] a significance could not be reproduced for patients with oligometastatic disease from EGAC. The results found in the available literature are highly heterogeneous, thus a clear recommendation of treatment for oligometastatic disease from EGAC is difficult to construct.

Limitations of this study included foremost the sample size of 48 heterogenous patients and the retrospective, non-randomized nature of this study. The patients analyzed in this manuscript are a highly selected collective of patients with metastasized EGAC, who underwent surgical resection. The majority of patients with metastatic disease received palliative chemotherapy only, and were not included in this study. Secondly, all types of metastases were included in our study, with some patients receiving additional HIPEC. There is a lack of a clear, agreed upon definition of oligometastases, thus a comparison within the literature is difficult. The inclusion of patients with peritoneal carcinomatosis could potentially skew the results. In single cases a misdiagnosis of preoperative cM1 status in patients with postoperative ypM0 status is possible and may thus create a bias. Furthermore, a multidisciplinary tumor board only selected patients with a good response to chemotherapy to proceed to surgery, for a potential cure of malignant disease.

## Conclusions

Although the current literature suggests improved overall survival for patients with perioperative FLOT and additional surgical resection of metastatic lesions, we could not establish a significant benefit for survival for patients undergoing additional resection of metastases compared to those, where only the primary tumor was resected. Oligometastatic EGAC is associated with overall poor survival rates, despite complete resection of all tumor manifestations. However, survival rates for patients with complete response after FLOT and surgical resection match survival rates of patients without any metastatic disease at primary diagnosis. The results from RCTs are needed to evaluate the significance of additional surgery for metastases, in order to define the best option for patients with oligometastatic disease in the era of multimodal treatment of EGAC.

## Data Availability

The datasets used and analyzed during the current study are available from the corresponding author on reasonable request.

## Abbreviations

FLOT: Chemotherpapy consisting of Docetaxel, Oxaliplatin, Leucovorin and 5FU
EGAC: Esophagogastric adenocarcinoma
RCT: Randomized controlled trial
ECF: Chemotherapy consisting of Epirubicin, Cisplatin and 5FU
ECX: Chemotherapy consisting of Epirubicin, Cisplatin and Capecitabin
HPR: Histopathological regression
GI: Gastrointestinal
CT: Computer tomography
PET-CT: Positron emission tomography
AEG: Esophagogastric junction cancers
HIPEC: Hyperthermic intraperitoneal chemotherapy
